# Impact of Stimulus Features on the Performance of a Gaze-Independent Brain-Computer Interface Based on Covert Spatial Attention Shifts

**DOI:** 10.3389/fnins.2020.591777

**Published:** 2020-12-01

**Authors:** Christoph Reichert, Igor Fabian Tellez Ceja, Catherine M. Sweeney-Reed, Hans-Jochen Heinze, Hermann Hinrichs, Stefan Dürschmid

**Affiliations:** ^1^Department of Behavioral Neurology, Leibniz Institute for Neurobiology, Magdeburg, Germany; ^2^Center for Behavioral Brain Sciences, Magdeburg, Germany; ^3^Research Campus STIMULATE, Magdeburg, Germany; ^4^Institute for Medical Engineering, Otto-von-Guericke University, Magdeburg, Germany; ^5^Department of Neurology, Otto-von-Guericke University, Magdeburg, Germany

**Keywords:** visual spatial attention, brain-computer interface, stimulus features, N2pc, canonical correlation analysis, gaze-independent, BCI

## Abstract

Regaining communication abilities in patients who are unable to speak or move is one of the main goals in decoding brain waves for brain-computer interface (BCI) control. Many BCI approaches designed for communication rely on attention to visual stimuli, commonly applying an oddball paradigm, and require both eye movements and adequate visual acuity. These abilities may, however, be absent in patients who depend on BCI communication. We have therefore developed a response-based communication BCI, which is independent of gaze shifts but utilizes covert shifts of attention to the left or right visual field. We recorded the electroencephalogram (EEG) from 29 channels and coregistered the vertical and horizontal electrooculogram. Data-driven decoding of small attention-based differences between the hemispheres, also known as N2pc, was performed using 14 posterior channels, which are expected to reflect correlates of visual spatial attention. Eighteen healthy participants responded to 120 statements by covertly directing attention to one of two colored symbols (green and red crosses for “yes” and “no,” respectively), presented in the user’s left and right visual field, respectively, while maintaining central gaze fixation. On average across participants, 88.5% (std: 7.8%) of responses were correctly decoded online. In order to investigate the potential influence of stimulus features on accuracy, we presented the symbols with different visual angles, by altering symbol size and eccentricity. The offline analysis revealed that stimulus features have a minimal impact on the controllability of the BCI. Hence, we show with our novel approach that spatial attention to a colored symbol is a robust method with which to control a BCI, which has the potential to support severely paralyzed people with impaired eye movements and low visual acuity in communicating with their environment.

## Introduction

A brain-computer interface (BCI) that can be controlled independently of gaze shifts could constitute a helpful assistive device for persons who suffer from severe neurological disorders. However, most developments in the field of BCI presume that the users can move their eyes. One of the most extensively studied brain signals is the steady-state-visual-evoked potential (SSVEP; Müller-Putz et al., 2005; Lin et al., 2007; Vialatte et al., 2010; Zhu et al., 2010), because its signal-to-noise ratio is relatively high, and low training effort is required to set up the decoder. It is commonly used in overt BCI control, since during covert attention paradigms, the behavioral performance, the SSVEP amplitude and BCI accuracy are comparatively reduced (Kelly et al., 2004; Walter et al., 2012). Another prominent example is the matrix speller, which was initially introduced by Farwell and Donchin (1988) and utilizes the P300 response to detect the time point at which the stimulus is presented at the target symbol location, on which the users’ attention is focused. Overt attention has also been shown to enable more reliable control than covert attention using matrix spellers (Brunner et al., 2010; Treder and Blankertz, 2010). Reliability is greater during overt compared with covert attention due to the additional modulation of early visual event-related potential (ERP) components according to the focus of attention (Treder and Blankertz, 2010; Frenzel et al., 2011), which is deemed to result from greater central than peripheral visual acuity. Hence, paradigms have been developed, which make use of more centrally located presentations (Treder et al., 2011b). For example, rapid serial visual presentation has been applied to detect a target in a series of rapidly presented symbols (Acqualagna et al., 2010; Lin et al., 2018). The disadvantage of this paradigm is that target presentations could be missed due to the attentional blink (Raymond et al., 1992). Moreover, in general, vision-based BCIs require good visual acuity, even if they are gaze-independent, but potential users frequently suffer from impaired vision (Halder et al., 2016). For this reason, auditory (Kübler et al., 2009; Halder et al., 2010, 2016; Hill et al., 2014) and tactile variants (Brouwer and van Erp, 2010; Jin et al., 2020) of the oddball paradigm have been investigated, with the finding that they provide inferior performance compared to BCIs based on visual stimuli (Severens et al., 2014).

In summary, a great deal of research into BCIs is dependent upon participants’ ability to execute eye movements. This requirement, however, largely neglects the fact that the main aim in BCI development is to provide a means of communication and control for patients in whom the ability to execute eye movements is impaired. Recently, it has been shown that spatial attention to peripherally presented colored stimuli permits reliable, gaze-independent control of a four class BCI (Reichert et al., 2020a). The paradigm takes advantage of the fact that shifts in attention to targets that pop up in the periphery of the visual field evoke slight interhemispheric differences in brain activity, depending on the side where the target was presented. This phenomenon has been intensively investigated in visual search experiments, where targets were presented in a search display among distractors, e.g., (Heinze et al., 1990; Luck and Hillyard, 1994a; Luck and Ford, 1998). Specifically, it has been found that in parieto-occipital regions contralateral to the presented target, 180–300 ms after onset, a stronger negative deflection compared to ipsilateral sites can be measured with the electroencephalogram (EEG). This component is known as the N2pc (Luck and Hillyard, 1994b) and is assumed to reflect the attentional selection of target features (Eimer, 1996). The fact that paying attention to simple features like color evokes spatially different ERPs depending on the visual hemifield where it was presented, suggests that the N2pc may be suited to gaze-independent BCI control. However, the potential advantages of using shifts in spatial attention have not yet been systematically evaluated for use in BCI control. The ability to classify several positions of peripherally presented targets has been evaluated using alpha activity (van Gerven and Jensen, 2009; Treder et al., 2011a) and ERPs (Fahrenfort et al., 2017). Classification of hemispheric differences, depending on the hemifield in which the target was presented, has been successfully applied for target detection in aerial images (Matran-Fernandez and Poli, 2017), for the detection of the tilt of Gabor patches (Xu et al., 2016) as well as in visual search for colored digits (Awni et al., 2013) and circles (Tian et al., 2019). While data in these studies were analyzed offline, to our knowledge, only one study has implemented a gaze-independent closed-loop BCI based on N2pc detection (Reichert et al., 2020a), where participants performed a two-dimensional navigation task. Here we extend this initial work to evaluate how stimulus size and eccentricity modulate the N2pc, which could alter the accuracy of the BCI. Specifically, we implemented a BCI for binary communication, suitable for responding to dichotomous questions. We hypothesize that hemispheric differences related to spatial attention are largely independent of stimulus size. This would permit the BCI to be operated with relatively large stimuli such that patients with low visual acuity can control the system. While the potential role of distractors in the composition of the N2pc has been investigated in a number of studies, with conclusions remaining controversial (Luck et al., 1997; Hopf et al., 2002; Hickey et al., 2009; Mazza et al., 2009), there have been no systematic investigations of the impact of symbol size and only one recent study exploring the impact of target eccentricity on the N2pc component (Papaioannou and Luck, 2020). However, BCI accuracy might depend on stimulus features, as such a dependency has been revealed using BCIs based on the P300 potential. For instance, accuracy was increased when faces were presented as stimuli as opposed to character flashes or meaningless images (Kaufmann et al., 2011), when luminance and chromatic features were combined (Takano et al., 2009) and when 3D stereo visual stimuli were presented as opposed to 2D stimuli (Qu et al., 2018). In contrast, symbol size and inter-symbol distance appears to have no general effect on the performance (Salvaris and Sepulveda, 2009). In the BCI experiment presented here, we varied symbol sizes and eccentricities to investigate whether such stimulus features have an impact on classification accuracy, and if so, to determine the optimal set of stimulus features to prevent poor performance due to inappropriate parameter choices in future studies.

## Materials and Methods

### Participants and Recordings

Eighteen healthy participants (10 female, 19 to 38 years, mean age: 27 years) took part in the study. All participants had normal or corrected to normal vision and reported no neurological impairment. They gave written informed consent and were paid for their participation. The study was approved by the Ethics Committee of the Otto-von-Guericke University, Magdeburg.

Participants were seated in an acoustically shielded and dimly lit cabin and viewed a 24” display (ASUS VG248QE) from a distance of 70 cm. Visual stimuli were registered by a photodiode to synchronize screen events with the EEG. The EEG was recorded from 29 Ag/AgCl electrodes, placed at standard positions of an extended 10–20 montage, using a BrainAmp DC Amplifier (Brain Products GmbH, Germany). Electrode measurements were referenced against the right mastoid and sampled at 250 Hz. Furthermore, the vertical and horizontal EOG (hEOG) was recorded simultaneously to register eye movements. Parallel to the recordings, EEG signals were transferred through TCP/IP to the BCI client.

### EOG Calibration

Before the experiment started, we recorded the EOG while participants were presented with a cross which they were asked to track with their gaze, and which changed its position every 1,250 ms. The position displacement relative to the center varied from 1 degree to 7 degrees horizontally and in 30% of trials we additionally displaced the cross by 2 degrees of visual angle vertically. Three times the cross was replaced by a circle, and participants were asked to perform an eye blink immediately. In total, 40 gaze shifts and three blinks were performed in an unpredictable order, resulting in approximately 1 min of EOG calibration. This procedure provided us with calibration data which characterize the strength of EOG signals as a function of gaze shift angle. We used these data to evaluate the degree of unintentional eye movement during BCI control.

### Stimulus and Task

**FIGURE 1:**
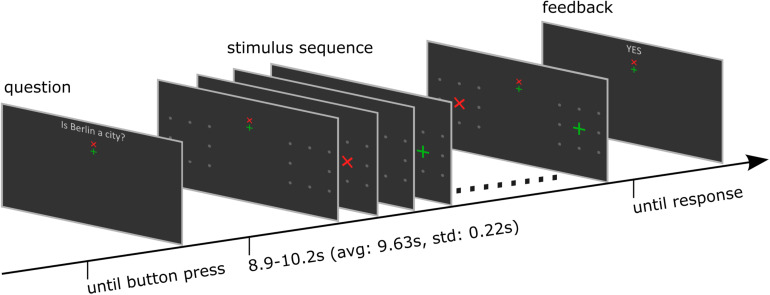
Structure of one trial. The trial started with a dichotomous question. The participant started the stimulation sequence by button press. After the stimulus presentation was finished, the BCI determined the target symbol, and the corresponding feedback was presented. Finally, participants indicated its correctness by responding with a further button press. See also the Supplementary Material for a video demonstration.

Participants were asked to respond to yes/no questions or statements by shifting their attention to a green +-cross to respond with “yes” or to a red × -cross to respond with “no” (see Figure 1). The first 96 questions and statements could be objectively answered with “yes” or “no,” e.g., “Is Berlin a city?”. To reduce the probability that there is a bias toward one particular answer, each question or statement also had a counterpart (e.g., “Is Berlin a continent?”), such that the numbers of expected “yes” and “no” answers were balanced. The last 24 questions could only be answered by the participant subjectively (e.g., “Are you a vegetarian?”), which constituted a demonstration of real-world application. Note that a correct answer was not relevant for the BCI. The BCI only evaluated the attentional shift, as decoded from the EEG and fed the result back to the participants. In turn, participants evaluated whether their intended response and the BCI feedback matched. A button press with the index finger indicated correct BCI feedback and a button press with the middle finger indicated that the feedback was not correct. Sixteen participants were native German speakers and were presented with questions in German. Two participants were not German but fluent in English, and we presented the same questions in English.

**FIGURE 2:**
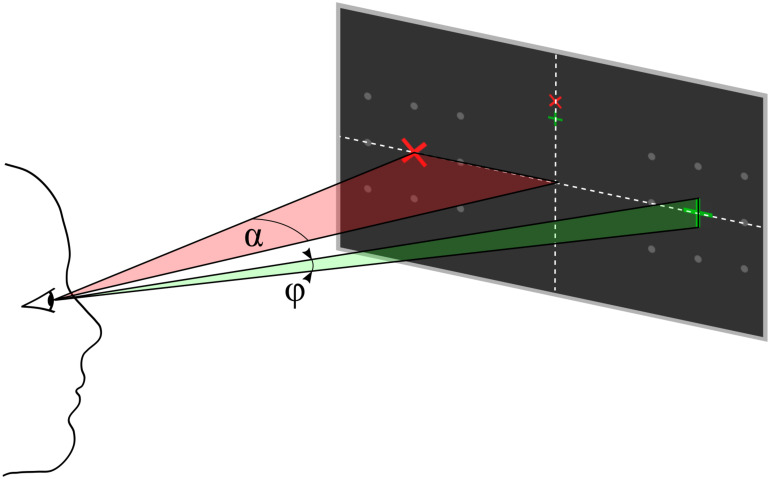
Parameter definition for eccentricity α and symbol size φ, both defined as visual angles. The participant’s gaze is directed to one of the upper central crosses.

Each trial started with presentation of a question. The participants had time to select the response and direct their gaze on the upper or lower but central fixation cross corresponding to the answer until they pressed a button to start the stimulus sequence. The differently colored fixation crosses were presented to help the participant to keep the target in mind during the entire trial. For example, if the participants decided to answer with yes, they directed their gaze to the green central fixation cross and focused their attention on the green +-cross, which was presented randomly left or right, during the whole stimulus sequence that followed. A single stimulus sequence comprised a series of ten stimuli, which was found previously to provide a good trade-off between stimulation time and accuracy (Reichert et al., 2020a). A single stimulus display thus consisted of a red × -cross presented in the left or right visual field and a green +-cross presented at the opposite visual field. The position at which the cross symbols appeared was surrounded by 8 gray dots, which were uninterruptedly presented throughout the whole trial to indicate the position where the stimulus would appear. In each stimulus display, we presented both a red and a green cross, one to the left and one to the right. Across the stimulus sequence, the colors were pseudo-randomly allocated to visual fields, with the restriction that the number of left/right presentations was balanced for both colors and that the same color was presented in the same visual field in a maximum of three consecutive stimulus displays. Each stimulus display was presented for 250 ms with a stimulus onset asynchrony of 850 ms, jittered by 0–250 ms. For an example stimulus sequence see the Supplementary Material. The horizontal position α and size of the stimuli φ was constant within a trial but varied between trials. We used four different φ levels, i.e., four symbol sizes which we define in visual angles (φ_1_ = 0.45°, φ_2_ = 0.90°, φ_3_ = 1.36°, and φ_4_ = 1.81°) and five different α levels, i.e., five eccentricities which we define in visual angles (α_1_ = 4°, α_2_ = 5.5°, α_3_ = 7°, α_4_ = 8.5°, and α_5_ = 10°). See also Figure 2 for a definition of parameters α and φ. We combined parameters φ_j_ with α*_j_* and with α*_j_*_+__1_, *j* = 1*…*4, such that 8 parameter pairs were tested. Each parameter set was applied in random order, three times per block, resulting in 24 trials per block. After the stimulus sequence was presented, feedback “yes” or “no” as decoded with the BCI, was presented. The participants confirmed the correctness of the feedback as described above. The resulting response was considered the ground truth, which we used to train and evaluate the BCI.

The first two blocks were conducted to acquire data to train the classifier. Therefore, we did not present questions in those blocks but instructed the participant to shift attention to the green symbol in the first block and to the red symbol in the second block. Afterward, we presented five blocks with 24 questions or statements each, except for one participant who performed only three question blocks due to technical issues. The classifier was initially trained with data from the first two blocks and retrained after each trial.

### Processing of EEG Data

In order to prevent hemispheric differences induced by a unilateral reference electrode, we re-referenced the EEG data to the average of left and right mastoid. Although we recorded 29 channels to provide full head coverage as open data (Reichert et al., 2020b), we used only 14 parieto-occipital channels (O9, O10, CP1, CP2, Pz, P3, P4, P7, P8, PO3, PO4, PO7, PO8, and Oz) to decode the shifts in visual attention. The EEG data corresponding to a stimulus sequence of a trial were cut out according to the start and stop events that we sent as trigger signals to the EEG device before and after presentation of the stimulus sequence. A 4th order zero-phase IIR Butterworth bandpass filter between 1.0 and 12.5 Hz was applied to the data, which were then resampled to 50 Hz. The stimulus onsets were determined from the signal sent by the photodiode. Afterward, the time series data were epoched starting from stimulus onset to 750 ms after stimulus onset. Since this resulted in 38 sampling points involving 14 channels, we can write an epoch as a matrix *X*_i_ ∈ *ℝ*^38×14^. Since the epochs of one trial, represented by a sequence of ten stimuli, refer to the same target item, data of one trial are composed of ten epochs. Epochs in which the green symbol was presented on the left and the red symbol on the right were labeled with *y*_i_ = 1, while epochs in which the red and green symbols were presented the other way around were labeled with *y*_i_ = −1.

### Decoding Approach

In this section we first describe the estimation of the decoding model, which is performed each time the classifier is trained – online and during the folds of cross validation. Afterward, we describe how to apply this model to unseen data – both to present online feedback and also to decode left-out trials in a cross validation. An implementation of the decoding approach can be found in the publicly available data set (Reichert et al., 2020b).

#### Model Estimation

We use canonical correlation analysis (CCA) to estimate spatial filters and canonical components from training data. The use of CCA has been proven efficient in the past for decoding SSVEPs (Nakanishi et al., 2015) and ERPs (Spüler et al., 2014; Xu et al., 2018; Xiao et al., 2020). The approach presented here is derived from our previous work (Reichert et al., 2016, 2017) and closely related to the approach recently published (Reichert et al., 2020a). CCA successively determines coefficient vectors *a* and *b* that linearly combine two sets of variables *X* and *Y* such that the correlation of *Xa* and *Yb* is maximal:

(1) $$\left(u,v\right)=\underset{a,b}{\text{argmax}}\text{corr}\,\left(X\,a,Y\,b\right)$$

where *u* and *v* are the resulting canonical variables. In the present implementation, *X* represents the concatenation of EEG epochs and *a* serves as a spatial filter. To reveal the hemispheric differences that characterize the shift of attention to the left or right visual field, the difference wave following left target presentations and right target presentations is commonly computed. We model the difference wave by composing a matrix *Y* that is a concatenation of identity matrices *I* ∈ *ℝ*^38×38^ weighted with the labels *y*_i_, where *y*_i_*I* indicates that the participant paid attention to the green symbol and −*y*_i_*I* indicates that the participant paid attention to the red symbol. The *k*th column in the variable set *Y* represents the *k*th time point after stimulus onset in an epoch and can be considered a concatenation of impulse functions, i.e., a vector of zeros being at the *k*th sample 1 if the target was presented left and -1 if the target was presented right. With this matrix, the difference waves for each channel in *X*, composed of *n* epochs per hemifield of target presentation, can be easily calculated as $\hat{X}=n^{-1}\,Y^{T}\,X$. However, since we want to determine optimal spatial filters, we apply CCA to *X* and *Y* using the MATLAB^®^ function *canoncorr* of the Statistics and Machine Learning Toolbox^TM^. As a result, we reveal 14 vectors *a*, whose elements can be used as channel weights and 14 vectors *b*, whose elements depict the canonical difference waves. Since the canonical correlation decreases with each iteration of the CCA algorithm, we retain only vectors that achieve a significance level *p* < 0.1 according to the *canoncorr* function. This procedure is performed with an arbitrary training set of trials to estimate the weight vectors *a* and *b* needed to classify the attended symbol.

#### Decoding a Sequence of Attention Shifts

After we have determined *a* and *b* from training data, the target of a new sequence is detected as follows. We concatenate the epochs of the trial as *X*′ and concatenate the corresponding weighted identity matrix *y*_i_*I* as *Y*′, i.e., we initially assume that the green symbol was the target symbol. We then calculate the Pearson product-moment correlation ρ of *X*′*a* and *Y*′*b* for all vectors we retained after CCA and calculate the mean correlation $\bar{\rho }$. The stimulus sequences were designed such that the target randomly changed between the visual hemifields. Because there are only two alternatives, which were presented simultaneously on opposite sites, the sequence for red targets is the reverse of the sequence for green targets. Thus, if $\bar{\rho }>0$, the canonical difference waves of the EEG correlate with the canonical difference waves of the model functions corresponding to the sequence of green symbols, as assumed when modeling *Y*′, and indicating that the participant intended to respond “yes.” If $\bar{\rho }<0$, the canonical difference waves of the EEG correlate with the canonical difference waves of the model functions corresponding to the sequence of red symbols, and we present “no” as feedback.

### Evaluation of BCI Performance

During the experiment, we decoded all trials that followed the first two training blocks and presented the result as feedback. For this online decoding, we involved all available trials we had recorded by that time and did not discriminate between stimulus features. In contrast, we performed offline decoding by leave-one-out cross-validation (LOOCV) and determined decoding accuracies that can be achieved by varying stimulus features. To compare the outcomes using small training subsets with those achieved with larger training sets, we matched the sample sizes by random selection of trials from the larger training set. We repeated the random selection one hundred times and averaged the decoding accuracies achieved in the LOOCVs.

We performed ANOVA and paired Wilcoxon signed rank tests to evaluate the impact of stimulus features. To determine the chance level of the decoder empirically, we performed a permutation test. Specifically, we randomly permuted the labels “yes” and “no,” which also implies randomized “target left” and “target right” assignments and performed LOOCV. This procedure was repeated 500 times. Afterward, we determined the 95% confidence interval from the distribution of decoding accuracies.

### EOG Analysis

We pursued two strategies to evaluate a potential impact of eye movements on BCI performance. First, we applied our decoding approach to the EOG data that we recorded during the experiment and compared the accuracy achieved with that achieved with the parieto-occipital EEG. Second, we compared the EOG recorded during the experiment with the EOG calibration data that we recorded prior to the experiment. Therefore, we calculated the deflection of the hEOG as follows. The hEOG data were segmented according to the cue in the EOG calibration and each single stimulus in the BCI experiment, respectively. We involved a time interval of 750 ms length starting from the cue or stimulus onset and performed baseline correction according to the first 100 ms. Afterward, we selected the 25 highest absolute values across the interval, which corresponds to 100 ms of strongest hEOG deflection, and averaged these values.

## Results

### Online BCI Performance

**FIGURE 3:**
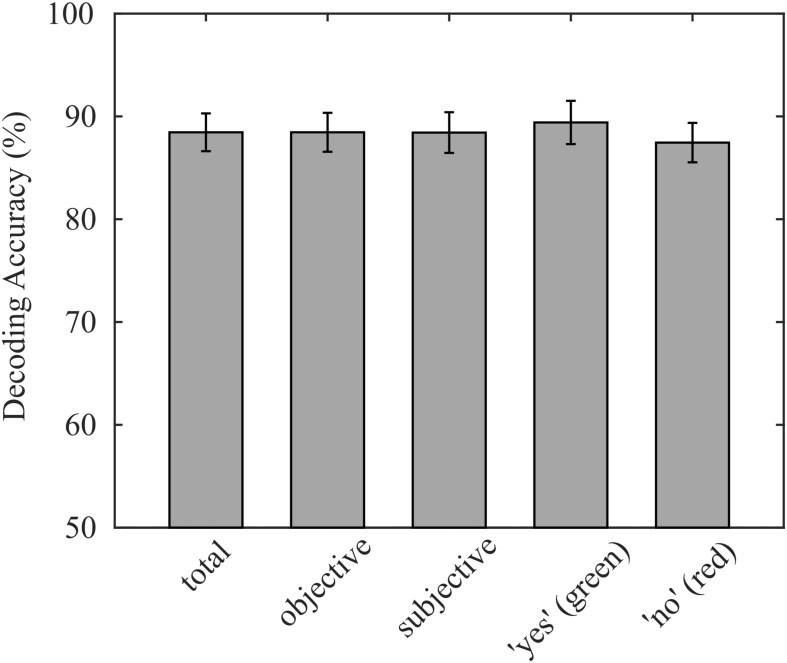
Average decoding accuracies achieved online, validated on the entire data set, separated for objective and subjective questions, and separated for green and red targets. No significant differences were found for any sub group.

On average, 88.5% (σ = 7.8%) of participants’ responses were correctly decoded with our decoding approach. Individual decoding accuracies ranged from 70.8% to 90.3%, while the chance level was 50%. The average accuracy corresponds to an information transfer rate of 3.02 bit/min, neglecting the time for asking questions and providing feedback. The decoding accuracy of questions with subjective answers (μ = 88.4%, σ = 8.4%) did not significantly differ from decoding accuracies of questions with obvious answers (*p* = 0.905; μ = 88.5%, and σ = 8.0%). Because we presented the same number of “yes” and “no” questions with objective answers in each run except in the last run, where answers were initially unknown, the sample sizes of the two classes were balanced, which reduces the probability that class sizes bias the classification. Consequently, the true positive rate of both classes was not statistically different (*p* = 0.298; “yes”: μ = 89.4%, σ = 8.9%; “no”: μ = 87.5%, and σ = 8.1%), indicating that the decoder was not biased. The reported decoding accuracies are summarized in Figure 3.

During the experiment, we retrained the classifier after each trial starting from the third run to provide the maximum number of trials available for model estimation. In practical use, the retraining would not be possible since we would not know the ground truth of the user’s intention. Therefore, we estimated the accuracy that can be achieved with only two runs of training by repeatedly performing a LOOCV with matched sample size. On average, 85.3% (σ = 11.1%) of trials were correctly decoded, which demonstrates that the retraining of the classifier improved the overall performance of the BCI significantly (*p* < 0.05).

### Evaluation of Stimulus Features

**FIGURE 4:**
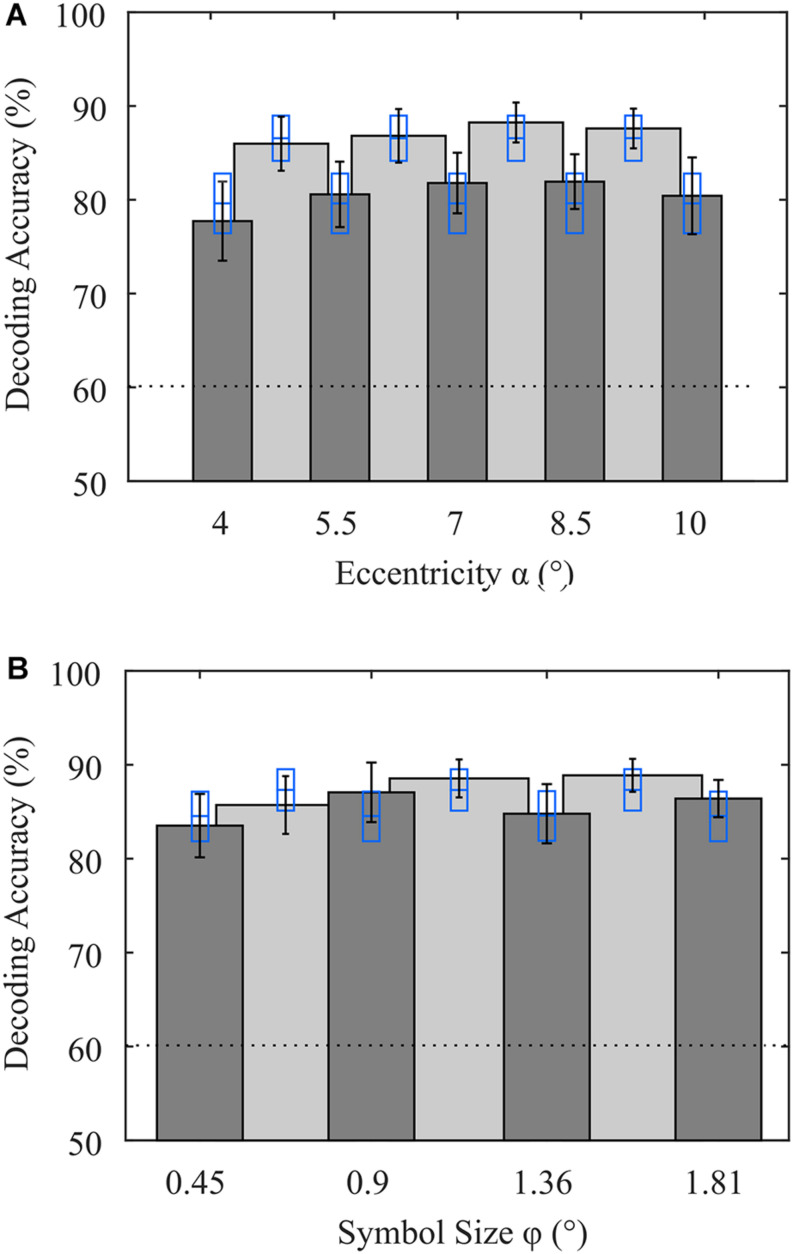
Average decoding accuracies achieved with variations in stimulus features **(A)** eccentricity and **(B)** symbol size. Dark gray bars indicate that only trials with that parameter (eccentricity or symbol size) were included in the analysis, but the other parameter varied (symbol size or eccentricity). Light gray bars indicate that in the analysis, trials associated with the two adjacent visual angles were included (e.g., 4° and 5.5°). Error bars indicate the standard error of the mean. Blue rectangles indicate the mean and the standard error of accuracies achieved with all stimulus features involved but sample sizes of the training data sets matched. The dotted line denotes the upper bound of the guessing level, as determined using a permutation test.

By using LOOCV, we maximize the amount of training data available for estimation of the spatial filters and the canonical difference waves required for detection of the attended symbol. We opted for LOOCV, because the number of samples available is small when validating subsets according to stimulus features. When all eccentricities and symbol sizes were included, as was the case for the online decoding, we achieved an average decoding accuracy of 88.6% (σ = 8.1%) using LOOCV. With this full data set, we performed a permutation test for each participant, which resulted in an upper threshold of 59.9% (σ = 0.8%) on average for the chance level. To investigate the impact of the stimulus’ eccentricity, we performed LOOCV involving only trials where stimuli were presented at a specific visual angle, irrespective of symbol size. Likewise, we performed the same analysis for the stimulus feature symbol size. To prevent bias in the evaluation of the performance of a subset due to larger sample sizes in the training data, we matched the sample sizes as described in (2.6). As a result, we found that, on average over participants, the eccentricities α_1_ = 4° and α_5_ = 10° resulted in slightly lower decoding accuracies, but there was no significant difference between visual angles α (Figure 4A). For the symbol size feature, there was also no statistically significant difference (Figure 4B). Furthermore, to increase the sample size of the training data set, we grouped each visual angle with its adjacent visual angle. While the accuracies were generally higher with these larger data sets, presumably due to a better generalizable model estimation, decoding accuracies did not statistically differ between tested visual angles. The symbol size groups φ_2_/φ_3_ and φ_3_/φ_4_ achieved statistically significantly higher accuracies compared to φ_1_/φ_2_ (*p* < 0.05, uncorrected). When comparing the parameter subsets, involving all trials but with matched sample size, only α_3_ = 7° and α_3_/α_4_ resulted in statistically significantly higher accuracies.

Finally, we also evaluated the performance of each parameter pair used in the experiment. Note that only 3 trials per run were available for each pair, resulting in 21 trials available for LOOCV. A 2-way ANOVA revealed no significant effect of the factors *eccentricity* (*p* = 0.98, *F*_4,143_ = 0.1) or *symbol size* (*p* = 0.72, *F*_3,143_ = 0.45). In Table 1, we show the results of the single parameter combinations. None of the combinations was significantly superior to another combination. Only the parameter combination (φ_3,_ α_3_) achieved statistically significant higher decoding accuracy (*p* < 0.05, uncorrected) compared to classification independent from stimulus features with matched sample size. Each of the combinations achieved individual maximum decoding accuracy in at least two participants. From the distribution of classification results independent from stimulus features with matched sample size, we repeatedly draw 8 decoding accuracies (in analogy to the 8 combinations) and determined the average of the maxima. This simulates the probability that a maximum value was achieved by an advantageous drawing of trials. We found no significant difference between maxima achieved with parameter combinations and maxima achieved by randomly drawing eight trial subsets from the entire data set. All these results indicate that the differences in stimulus features chosen in this study have no significant impact on the decoding accuracy achieved with the spatial attention paradigm.

**TABLE 1:** Decoding accuracy achieved with single parameter pairs (standard deviation in parenthesis).

Visual angle	α_*1*_	α_*2*_	α_*3*_	α_*4*_	α_*5*_
φ_1_	77.7 (17.9) %	79.3 (16.9) %	–	–	–
φ_2_	–	83.2 (13.0) %	82.5 (15.4) %	–	–
φ_3_	–	–	79.6 (15.5) %	79.7 (14.6) %	—
φ_4_	–	–	–	83.4 (17.6) %	80.4 (17.4) %

### Impact of Eye Movements

**FIGURE 5:**
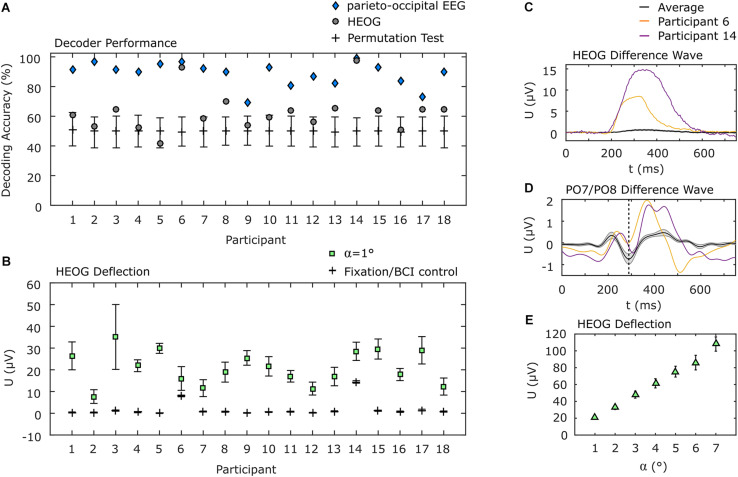
EOG analysis. **(A)** Individual decoding accuracies achieved with EOG and with parieto-occipital EEG. EOG decoding accuracies for participants 6 and 14 were above 90% but below those achieved using EEG. Error bars indicate the 95% confidence interval of a permutation test. **(B)** Individual horizontal EOG deflections. Here the same two participants also show higher deflections during BCI use, but they are still below the deflection measured when 1° movements were requested in a calibration procedure. All other participants show almost no deflections during BCI use. **(C)** EOG difference wave between left and right target presentation. Participants 6 and 14 show considerably higher EOG deflection compared to the average. **(D)** PO7/PO8 difference wave. Participants 6 and 14 show the N2pc component (dashed vertical line), typically evoked during spatial attention shifts, similar to the average but with higher positive deflection afterward. **(E)** Deflection of the EOG as a function of the angle of gaze shift during EOG calibration. Error bars in **(B,E)** indicate the standard error of the mean.

The use of only parieto-occipital electrodes reduces the probability that eye movements have a systematic impact on the decoding accuracy. However, we pursued two additional strategies to explore a potential impact of eye movements. Firstly, if eye movements nonetheless played a substantial role in discrimination of visual attention shifts, decoding the EOG data should result in higher accuracies than the EEG channels over brain areas attributed to visual processing. Therefore, we applied the same decoding approach to the EOG signals as we applied to the parieto-occipital EEG channels. The average decoding accuracy of 63.0% (σ = 13.6%) is significantly lower than that achieved using EEG data (*p* < 0.001). Notably, for two of the participants, classification of EOG data resulted in an accuracy of above 90%, but the EEG accuracy was higher still for those participants (see Figure 5A for details). For the remaining participants, EOG decoding accuracy was within or slightly above the confidence interval for chance as determined by a permutation test.

In a second analysis, we determined the deflection of the hEOG during the experiment, according to the presentation side of the target, and compared it with the hEOG deflection obtained from defined eye movements. We found that most of the participants showed almost no hEOG deflection. Specifically, it was much lower than the smallest gaze angle of 1°, which we tested in the EOG calibration session. This finding is in concordance with the results obtained applying our decoding approach to EOG data only, leading to accuracies close to the guessing level for most of the participants. The two participants who showed high decoding accuracy based on EOG channels also showed highest hEOG deflections, but still below that of 1° gaze angle (Figure 5B). To further provide evidence that the BCI was not influenced by eye movements, even in the two participants showing higher EOG deflections compared to the remaining participants, we show the difference waves (ipsilateral target presentation subtracted from contralateral target presentations) of the hEOG (Figure 5C) and of the EEG signal at PO7/PO8 (Figure 5D) for these participants and compare it with the average signals from the remaining participants. While the hEOG was much larger in these two participants compared to the group average, the difference wave at PO7/PO8 shows the typical N2pc component around 288 ms, which is a marker for shifts in spatial attention. However, the eye movements also might propagate to these channels as indicated by the larger positive deflection. Finally, we show the hEOG deflections as a function of the gaze angle obtained in an EOG calibration session in Figure 5E. Comparison indicates that if participants had shifted their gaze directly to the target with the lowest eccentricity (α_1_ = 4°), an average hEOG deflection of 61.4 μV (σ = 23.2 μV) would be apparent. However, the average deflection during the BCI experiment was much lower (<1 μV for 13 participants, <1.5 μV for 3 participants, <8 μV for 1 participant and <15 μV for 1 participant).

## Discussion

The BCI implementation presented here, demonstrates that questions can reliably be answered with “yes” or “no” simply by directing visual spatial attention to one of two simultaneously presented colored symbols. Sensitivity to differences in stimulus features, specifically to the size and eccentricity of presented symbols, could not be found with statistical evidence. However, large symbols tended to lead to more accurate decoding, which suggests that even for persons with impaired vision, attention to a perceived color in the left or right visual hemifield might be sufficient to determine the shift of spatial attention for reliable communication.

Although the information transfer rate of binary classification is low by definition, communication on a “yes” or “no” basis could provide important assistance in maintaining social interaction for persons who cannot otherwise communicate. The fact that the BCI can be controlled independently of gaze shifts and is thus potentially accessible to severely disabled potential users may be deemed to compensate for the low information transfer. Gaze-independent BCIs with two answer options have indeed been implemented using several other modalities. For example, the covert shift of attention to auditory stimuli has been decoded using EEG-based BCIs, achieving a bit rate of 2.46 bit/min and an accuracy of 78.5% (Halder et al., 2010), which is below the performance achieved with our visual spatial attention approach. Another auditory approach achieved 4.98 bit/min neglecting inter-trial gaps and 85% and 77% accuracy, respectively, (Hill and Schölkopf, 2012; Hill et al., 2014). Using motor imagery of hand and foot movement to respond to auditorily presented questions, only two of ten healthy participants achieved effective control (Müller-Putz et al., 2013). In further studies, covert speech was performed in the form of mental repetition of the words “yes” and “no,” which resulted in decoding accuracies of 63.2% (Sereshkeh et al., 2017a) and 69.3% (Sereshkeh et al., 2017b). An independent BCI based on SSVEPs and a non-spatial visual attention paradigm has produced an accuracy of 72.6% (Zhang et al., 2010). A new vibrotactile stimulation paradigm achieved an accuracy of 76.7% and a bit rate of 1.35 bit/min in comparison to the benchmark paradigm where accuracy was 65.6% and the bit rate was 0.61 bit/min (Jin et al., 2020). Vibrotactile stimuli were also tested for communication in six locked-in syndrome patients (Lugo et al., 2014), where the grand average accuracy was reported to reach 55.3%. In a follow-up study, an 86.7% decoding accuracy was achieved with vibrotactile stimulation and 83.3% using motor imagery in healthy controls, but only 63.1% accuracy was achieved with vibrotactile stimulation and 58.2% with motor imagery in a patient group (Guger et al., 2017). This dramatic reduction in decoding accuracy was seen in patients suffering from a motor neuron disease when the somatosensory and motor cortex were involved in the control strategy, but it remains unclear whether such a reduction in accuracy would also be expected using visual spatial attention to provide responses. In a case study investigating the ability of a completely locked-in patient to communicate “yes” and “no” by thinking the answer, over 70% accuracy was achieved using functional near infrared spectroscopy (Gallegos-Ayala et al., 2014). This approach was further investigated with healthy subjects where an accuracy of 75% was achieved (Hwang et al., 2016). While there is no firm evidence that it is possible to discriminate in EEG signals between simply thinking “yes” or “no,” we have shown that the direction of visual spatial attention can be clearly discriminated, with a 88.5% decoding accuracy, on a binary basis in EEG recordings from healthy participants. Future studies are required to determine applicability in patient groups.

Importantly, the results of the current study suggest that the decoding of spatial attention shifts is largely independent of several empirically chosen parameters for stimulus presentation. The parameter choices were made according to commonly reported measures in N2pc-relevant literature (Luck and Hillyard, 1994b; Eimer, 1996; Luck et al., 1997; Hickey et al., 2009; Mazza et al., 2009; Grubert et al., 2017; Donohue et al., 2018; Drisdelle and Jolicoeur, 2019). The independence of BCI performance from stimulus features is indicated by the high accuracy achieved online, where we trained and tested the BCI using all trial types irrespective of the symbol size and eccentricity. However, since the number of samples available to train a classifier was higher for the whole data set than that available for subsets that represent specific stimulus features, the trained model might have been estimated with better generalizability, leading to a higher accuracy. We therefore reduced the number of samples used to train the classifier on data including all possible stimulus features to match the sample sizes of the subsets based on particular stimulus features. None of the subsets of trials associated with single parameters for stimulus features led to significantly different accuracy when averaged across participants but we found a marginal significance for the eccentricity 7 °. The combination of two subsets of adjacent parameter values increased the accuracy compared to single subsets but again, the major increase can be attributed to the greater number of samples. In a recent study, magnified and non-magnified symbols were presented at different eccentricities showing different amplitudes at different eccentricities, but no interaction was found between eccentricity and magnification (Papaioannou and Luck, 2020), i.e., the N2pc amplitude accompanying larger symbols did not differ from that observed when smaller symbols were employed. This lack of difference is in accord with our finding that alterations in symbol size did not result in a significant change in decoding accuracy, which makes the paradigm potentially suitable for persons who suffer from impaired vision, because the perception of the target color, as the to be attended feature, in the left or right visual field, independent of symbol shape, might be sufficient to decode the attention shift of those persons. Furthermore, visually impaired people might not be able to discriminate between different symbols in the same visual hemifield, which is why we did not present competing distractors, although they are assumed to increase relevant hemispherical differences in the EEG (Luck et al., 1997). In our experiment, participants achieved reliable control without presentation of competing distractors. Whether distractors could lead to a further increase in accuracy is one of the questions for future studies. Regarding eccentricity, there was trend in our data to better discrimination of spatial attention when targets were presented in the range of five to nine degrees visual angle. Papaioannou and Luck (2020) investigated the effect of eccentricities at visual angles smaller than 4° (which was the smallest in our study) and found that the N2pc amplitude was constant, even for stimuli near the midline, but the amplitude was significantly smaller at 8° visual angle. In contrast, the post-N2pc positivity was significantly larger at 8°. The difference in our findings could be explained by the fact that the algorithm we used automatically determined the relevant features from the EEG and thus, it is not clear whether the N2pc or the post-N2pc positivity is the main feature that discriminated between attention shifts with the different eccentricities. Further investigations are required in this regard. Also, the parameter space might be extended in future studies to determine individual boundaries at which the attentional shift is detectable from short sequences of stimuli. While decoding accuracy is reduced in visual P300-based BCIs with increasing eccentricity of the target (Treder and Blankertz, 2010) this study suggests that N2pc-based BCIs, which depend on shifted stimuli, are largely insensitive to eccentricity.

We evaluated the hEOG to exclude the possibility that eye movements have an impact on BCI control. For this purpose, we recorded defined saccades in a short session before the actual experiment. The hEOG amplitudes we recorded were in accordance with the findings of Lins et al. (1993). The hEOG activity we measured during BCI control was less in amplitude than measurements during execution of 1° saccades for all participants and close to zero for most of the participants. Only two participants unintentionally performed small saccades (below 1°) during BCI control, which might have biased the decoding accuracy, but their decoding accuracy using EEG was nonetheless greater than using EOG. However, since we involved only parieto-occipital electrodes showing the typical time course observed during visual spatial attention, it is unlikely that BCI control was achieved by eye movements.

The BCI we propose here is suited to communication of responses to yes/no questions simply by directing visual spatial attention to a colored, peripherally presented symbol in persons who are unable to move their eyes and has the potential to be used in the absence of high visual acuity. Our data suggest that the decoding accuracy of visual spatial attention is largely independent of symbol size and eccentricity. The new approach could potentially serve as an assistive communication technique for patients suffering from severe motor neuron diseases. Future work should involve evaluation of decoding accuracy in visually impaired individuals.

## Conclusion

We implemented a BCI that decodes binary decisions from a short series of ERPs that solely reflect processes of spatial attention. We found that the symbol size and eccentricity of the bilaterally presented stimuli have a minimal impact on the overall accuracy of the BCI. Consequently, attention to simple features like color, independent of the stimulus’ shape, might be sufficient to control such a BCI, rendering it promising for visually impaired end-users.

## Data Availability Statement

The datasets presented in this study can be found in the Open Science Repository of the Otto-von-Guericke University, Magdeburg (10.24352/UB.OVGU-2020-155) and in the BNCI Horizon 2020 web repository (http://bnci-horizon-2020.eu/database/data-sets).

## Ethics Statement

The studies involving human participants were reviewed and approved by Ethics Committee of the Otto-von-Guericke University, Magdeburg. The participants provided their written informed consent to participate in this study.

## Author Contributions

CR and SD designed the experiment. CR implemented the BCI. CR and IFTC collected the data. CR, IFTC, and SD analyzed the data. CR, SD, CMS-R, H-JH, and HH interpreted the data. CR, SD, and CMS-R wrote the manuscript. All authors contributed to the article and approved the submitted version.

## Conflict of Interest

The authors declare that the research was conducted in the absence of any commercial or financial relationships that could be construed as a potential conflict of interest.
